# Preliminary experience on laparoscopic pancreaticoduodenal combined with major venous resection and reconstruction anastomosis

**DOI:** 10.3389/fsurg.2022.974214

**Published:** 2022-09-08

**Authors:** Xuehui Peng, Yonggang He, Yichen Tang, Xiaomin Yang, Wen Huang, Jing Li, Lu Zheng, Xiaobing Huang

**Affiliations:** Department of Hepatobiliary, The Second Affiliated Hospital of Army Medical University, Chongqing, China

**Keywords:** laparoscopic pancreatoduodenectomy, pancreas mesangial resection, anastomosis, surgical approach, venous resection and reconstruction

## Abstract

**Objective:**

This study aims to summarize our experience in laparoscopic pancreatoduodenectomy (LPD) combined with major venous resection and reconstruction, as well as to evaluate its safety and discuss the surgical approach.

**Methods:**

We retrospectively analyzed 14 cases of patients diagnosed with pancreatic tumors invaded the superior mesenteric vein or portal vein who had undergone LPD combined with major venous resection and reconstruction in our center from May 2016 to May 2020. Clinical data of these 14 patients were collected and analyzed, including general information (age, gender, pathological diagnosis, body mass index, etc.), intraoperative data (operation time, intraoperative blood loss, transit rate, blood transfusion, tumor diameter, R0 resection rate, cleaning lymph node number, removal vessel length, venous reconstruction time), and postoperative results (gastrointestinal function recovery, postoperative hospital time, complications, and fatality rate). Patients were followed up after surgery, and data were collected for statistical analysis.

**Results:**

A total of 14 patients (9 males and 5 females) received LPD combined with major venous resection and reconstruction by arterial approach. The mean age was 52.5 (43–74) years old. Three of these 14 patients had routine wedge resection, 9 had opposite-to-end anastomosis after venous resection, 2 had artificial venous replacement, and the average length of removal vessel was 3.1 (2–4.5) cm. The operation time was 395 (310–570) min; the venous blocking time was 29.7 (26–50) min; the hospitalization stay was 13.6 (9–39) days. There was no grade B or C postoperative pancreatic fistula (POPF) that occurred, only one patient had biochemical fistula. One patient had upper gastrointestinal bleeding after subcutaneous injection of low molecular weight (LMW) heparin, and the condition was alleviated after conservative treatment, and one had pulmonary infection. The 12-month disease-free survival rate was 85.7%, and the 12-month overall survival rate was 92.8%. No patients had 30-day re-admission or death.

**Conclusion:**

On the basis of the surgeon’s proficiency in open pancreatoduodenectomy combined with venous resection and reconstruction and standard LPD, the arterial approach for LPD combined with major venous resection and reconstruction is safe and feasible.

## Introduction

Pancreatoduodenectomy (PD) is a classical surgical method for the treatment of benign and malignant diseases around the head or ampulla of the pancreas (1, 2). An international expert consensus (3) has been already published for the surgical treatment of tumors in the head of the pancreas with local invasion of the portal vein and superior mesenteric vein: for patients who were expected to achieve R0 resection with combined portal vein and superior mesenteric vein resection, PD combined with corresponding vein resection could not only improve R0 resection rate but also had no significant difference in complications, mortality, prognosis compared to PD with no-vascular-invasion. Several previous studies on combined arterial or major venous resection for the surgical treatment of pancreatic cancer had also supported it (4–6).

As emerging research studies uncovered, compared with open pancreaticoduodenectomy (OPD), LPD has advantages of smaller trauma, faster recovery, less bleeding, and higher postoperative quality of life for patients, and the safety and feasibility can be guaranteed by experienced pancreatic surgeons (7, 8). More recently, LPD combined with vascular resection and reconstruction has also been reported although it required professional surgical skills (9–12). This study aims to establish a safe and feasible method for resection and reconstruction of major venous combined with LPD based on our preliminary experience.

## Methods

### Study design and patients

This was a retrospective study. A total of 14 patients who had undergone LPD combined with major venous resection and reconstruction in the Department of Hepatobiliary, the Second Affiliated Hospital of the Army Medical University from May 2016 to May 2020 were included. We collected their general information (including age, gender, pathology diagnosis, BMI, etc.), intraoperative conditions (operation time, intraoperative blood loss, conversion rate of surgical way, blood transfusion, tumor diameter, R0 resection rate, amount of lymph node clearing, venous reconstruction time, etc.), and postoperative conditions (including gastrointestinal function recovery, postoperative hospital stay, complications, and death rate). Patients were followed up for up to 24 months after surgery. The surgeries were performed by the same surgeon; CT, MRI, and hepatic arteriovenous angiography were completed for each patient before operation to decide whether the tumor had a major artery invasion, the invading length of the vein, or any abnormal arteries. Patients were given preventive anti-infection, nutrition support, enzyme suppression, and other treatments after surgery. This study strictly follows the Declaration of Helsinki and the International Theoretical Guidelines for Biomedical Research Involving People and was submitted to the Medical Ethics Committee of the Second Affiliated Hospital of the Army Medical University for approval. Patients were required to sign informed consent to agree with the treatment.

The inclusion criteria are as follows: (1) preoperative imaging for diagnosis of pancreatic head tumor with no distant metastasis; (2) tumor diameter <5 cm, preoperative imaging indicated tumor invasion of portal vein or superior mesenteric vein, but without obvious stenosis or unilateral stenosis (involving vessel circumference diameter ≤1/3 and the invasion length <5 cm), without peripheral major artery invasion, and the invaded vessel could be completely resected and reconstructed; (3) good distal and proximal conditions for venous reconstruction, and patients with monotype SMV (unsuitable for patients with downward branch involvement of SMV); and (4) no serious multi-organ insufficiency such as the heart, lungs, kidneys or brain.

The exclusion criteria are as follows: (1) poor compliance and difficulty strictly adhering to the doctor's instructions; (2) serious basic comorbidities and inability to tolerate surgery after active adjustment; (3) tumors invaded major arteries such as peripheral hepatic artery and superior mesenteric artery or distant metastasis; and (4) lost to follow-up with incomplete perioperative data.

### Procedure of laparoscopic pancreaticoduodenectomy combined with major venous resection and reconstruction

**Figure 1:**
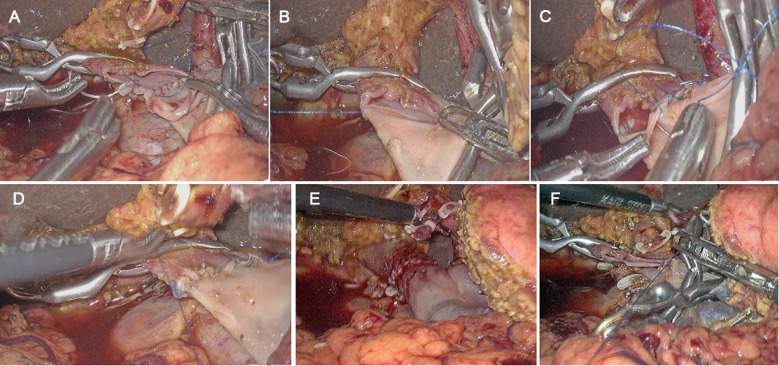
Anastomosis process of laparoscopic pancreatoduodenectomy (LPD) combined with major venous resection and reconstruction. (**A**) Interrupted suture on the left side of the extravascular and knotted; (**B**) continuous suture on the posterior wall of the blood vessel; (**C**) completion of continuous suture on the posterior wall; (**D**) continuous suture on the anterior wall; (**E**) completion of continuous suture on the anterior wall; (**F**) no stenosis of vessel after anastomosis.

The LPD procedure was referred to as the method that our group had published recently (13). The detailed process was as follows: first, the lower margin of the pancreas was dissected to expose the superior mesenteric vein (SMV), the Henle stem was broken, the SMV was isolated until the laparoscopic bulldog clamp can be used; and then, sweeping lymph nodes of group 14 v. Clamping both ends of the tumor-invading vessels with the bulldog clamp, removing the tumor and the invaded part of the vascular; the vessels were sutured by prolene line. The reconstruction method was determined by the degree of vascular invasion: (1) if the tumor only invaded the vascular wall, local venous wedge resection was performed; (2) if the tumor invades a wide range of vascular, tumor combined with segmental vascular resection and continuous end-to-end anastomosis or artificial vascular replacement was needed. And for vascular reconstruction, the 3 o’clock and 9 o’clock anastomosis method was used. Finally, fixed the vessel at one end and sutured to the other end using a 5–0 prolene line, and then sutured the anterior wall continuously (Figures 1A–E). After successful venous anastomosis, the portal vein was opened, and the vessels were unobstructed without bleeding or stenosis (Figure 1F). Digestive tract reconstruction was performed after venous resection and anastomosis, and a modified blumgart anastomosis was adopted for the pancreatic reconstruction (14). After the operation, one abdominal drainage tube was placed ahead of the pancreatic duct-jejunal anastomosis and one behind the bile duct-jejunal anastomosis, respectively.

### Artificial venous replacement

The artificial blood vessel is provided by the manufacturer of Gore (USA), which is made of polytetrafluoroethylene (PTFE), and the models are S0604, S0804, S1004, S1203, SA1403, SA1603, SA1803, SA2003, etc. The selection of the model depends on the diameter of the vein. The anastomosis method is to suture the anterior and posterior walls continuously with a 5–0 prolene line. The diameter of the artificial blood vessel is selected to match the diameter of the portal vein/superior mesenteric vein to avoid tension; and the length of the artificial blood vessel is almost the same as that of the removed blood vessel, which should not be too long or too short. For these LPD patients who had undergone artificial venous replacement, 4,000 u of LMW-heparin was administered by subcutaneous injection after surgery, once a day, and aspirin enteric-coated tablets with 100 mg/day were used after discharge.

### Postoperative management and definitions

All patients received prophylactic antibiotics perioperatively and underwent gastric tube extraction 2 days after surgery. All patients began to drink water after feeling hungry and entered a liquid diet after gas exhaustion. The drainage amylase was routinely examined on day 3 postoperation. The intraperitoneal drainage tube was removed when the drainage fluid was three times less than the normal value with the exclusion of abdominal bleeding (generally for an average of 5 days). Arteriovenous angiography was used in all patients on the fifth postoperative day to assess the degree of venous patency. All patients were treated with LMW heparin with 2,500–5,000 IU/day for 7 days in a row. For patients undergoing artificial venous replacement, LMW heparin was replaced with warfarin before discharge to maintain an international standardized ratio of 1.5:2.

Postoperative pancreatic fistula (POPF) was defined, according to the International Standard of Research Group of Pancreatic Fistula (ISGPF) (15), as the amylase content in abdominal drainage fluid was at least three times higher than serum amylase 3 days or longer after surgery. Gastric emptying disorder is defined, according to the ISGPS standard published in 2007 (16), as after excluding mechanical factors such as anastomotic obstruction by upper gastrointestinal angiography or gastroscopy, one of the following conditions occurred could be diagnosed: (1) the indwelling time of gastric tube was more than 3 days after surgery; (2) require re-insertion of the tube after extubation due to vomiting or other reasons; and (3) and unable to eat solid food 7 days after the operation. Surgical site infection (SSI): the diagnostic criteria were referred to the definition of the National Hospital Infection Surveillance System of the US Centers for Disease Control and Prevention (17). The postoperative complication grading adopted the Clavien–Dindo grading system in 2004 (18).

### Follow up strategy

Postoperative follow-up was performed by a combination of outpatient examination and telephone visits. Related data were recorded in the medical system of the Second Affiliated Hospital of Army Medical University. Disease-free survival and overall survival data of the patients were obtained by telephone visits.

### Statistical analysis

SPSS 26.0 (SPSS Inc., IBM, Armonk, NY, USA) statistical software was used for data analysis in this study. The measurement data were expressed by mean ± standard deviation in accordance with normal distribution and by median (quartile spacing). The survival curve was calculated and drawn by Kaplan–Meier method and GraphPad Prism (version 8.0.2).

## Results

In our retrospective study, the data of 14 patients (9 males and 5 females) were analyzed. The demographic characteristics of the patients were shown in Table 1. The mean age was 52.5 (43–74) years old, and the average body mass index was 22.5 (17.1–32.4) kg/m^2^. The pathological diagnosis results revealed that 13 of these patients had pancreatic cancer and one had cholangiocarcinoma.

**Table 1:** Patient characteristics data.

Characteristics	Results
Case number	14
Gender (male/female)	9/5
Age (year)	58.5 (13–80)
BMI (kg/m^2^)^a^	20.9 (16.23–30.9)
American Society of Anesthesiology	
I	9
II	4
III	1
Disease	
Cancer of pancreas	13
Cholangiocarcinoma	1

^a^ Data are expressed as median and inter-quartile range.

BMI, body mass index.

Among these 14 patients, 2 had received neoadjuvant therapy (AG regimen: gemcitabine 1,000 mg/m^2^ plus nab-paclitaxel 125 mg/m^2^ on days 1, 8, and 15, every 4 weeks). After three rounds of therapy, tumor evaluations were made, and when the tumor shrunk and the tumor stage decreased, an operation was performed.

Intraoperative conditions were shown in Table 2. All patients had LPD, three of them had wedge resection, nine cases had opposite-to-end anastomosis after venous resection; two cases underwent artificial venous replacement. The average removal vessel length was 3 (2–4.5) cm. The operation time was 395 (310–570) min, the mean vascular blocking time was 29.7 (26–50) min, and the intraoperative bleeding loss was 464 (200–1,000) ml.

**Table 2:** Intraoperative conditions.

Intraoperative situation		Results
Vascular resection	Portal vein	9
	Venae mesenterica superior	5
Resection/reconstruction method	Segmental/end-to-end anastomosis	9
	Wedge/local suture	3
	Segmental/artificial vessels	2
Vasculectomy length (cm)	Wedge	0
	Segmental resection	3.1 (2–4.5)
Operation time (min)^a^		395 (310–570)
Blocking time (min)^a^		29.7 (26–50)
Intraoperative bleeding (ml)^a^		464 (200–1,000)
Transfer rate (case)		0
Lymph node	Number of cleaning lymph nodes	18 (16–24)
	Positive rate (*n*%)	4.1%

^a^ Data are expressed as median and inter-quartile range.

The postoperative results were summarized in Table 3. The mean postoperative hospitalization stay was 13.6 (9–39) days. Only one patient had biochemical fistula, no grade B or C POPF occurred. One patient had upper gastrointestinal bleeding after subcutaneous injection of LMW heparin, and the condition was relieved after conservative treatment, and one had pulmonary infection. Postoperative complications occurred in three cases (21.3%), including one case (7.1%) of postoperative gastrointestinal bleeding, one case of biochemical fistula (7.1%), and one case of pulmonary infection (7.1%), the remaining patients had no intra-abdominal hemorrhage, biliary fistula, celiac leakage, abdominal infection, abdominal effusion, gastric emptying disorder, or other complications. The patient with gastrointestinal hemorrhage experienced melena was attributable to dosing LMW heparin after artificial venous replacement, and the symptom was relieved after conservative medical treatment. The patient with pulmonary infection due to a history of COPD and the patient refused to get out of bed after operation. A chest radiograph indicated infection in the right lower lung, which improved after treatment with nebulized expectorants and anti-infection. One case of biochemical fistula was recovered and discharged after conservative unobstructed drainage treatment.

**Table 3:** Postoperative results.

Postoperative information	Results
Postoperative hospital stay time (day)	13.6 (9–39)
Complications	
Hemorrhage	1 (7.1%)
Hemorrhage of digestive tract	1 (7.1%)
Intraperitoneal hemorrhage	0
Postoperative pancreatic fistula	
Biochemical fistula	1 (7.1%)
Grade B	0
Grade C	0
Bile leakage	0
Celiac leakage	0
Pulmonary infection	1 (7.1%)
Abdominal infection	0
Gastric emptying disorder	0
Ascites	0

**Figure 2:**
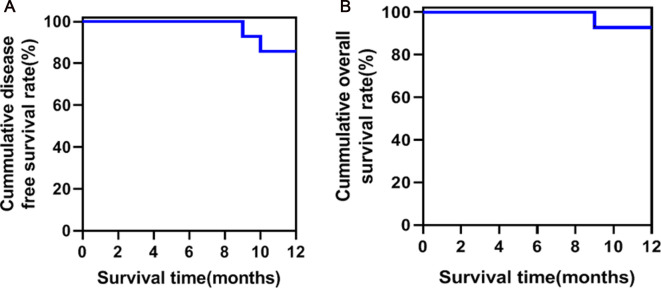
Survival curve of patients with laparoscopic pancreatoduodenectomy (LPD) combined with major venous resection to reconstruction. (**A**) Disease-free survival curve; (**B)**. overall survival curve.

All patients were regularly followed up in outpatient visits or telephone visits. The average follow-up time was 18.5 (15–24) months. Five patients refused chemotherapy for worrying about its side effects, and two patients were unable to tolerate standard chemotherapy. Finally, seven patients received systemic chemotherapy with gemcitabine (1,000 mg/m^2^, day 1, 8, 15, every 4 weeks a course, and six courses each cycle). The 12-month disease-free survival rate and overall survival were shown in Figure 2. One patient died of renal failure and two patients developed liver metastasis during the follow-up period. No patients had 30 days of re-admission or death, and no patients had complications during the 15–24 months of follow-up.

## Discussion

Radical resection (R0) of pancreatic cancer is the most effective way that may cure, prolong the survival and improve the life quality of pancreatic cancer patients without distant metastasis (19–21). For advanced pancreatic cancer with tumor invaded to the portal vein or superior mesenteric vein, PD combined with vascular resection and reconstruction is performed, and the safety and feasibility of OPD have been verified (22). With the improvement of laparoscopic instruments, the improvement of surgeons’ clinical skills, and perioperative clinical processing experience, LPD is gradually popularized. Studies have confirmed the safety and effectiveness of LPD in larger pancreatic centers (3).

Due to the complicated and difficult operation of PD combined with vascular resection and reconstruction under laparoscopy, there is little research reported about the safety or feasibility of LPD combined with vascular resection in the treatment of pancreatic cancer. Until 2011, Kendrick et al. (11) and Giulianotti et al. (23) first reported LPD combined with PV-SMV vascular resection and reconstruction and Da Vinci Robotic pancreaticoduodenectomy (RPD). Croome et al. (10) compared 31 cases of LPD and 58 cases of OPD from Mayo Center and suggested that under the premise of mastering standard LPD techniques and adept vascular anastomosis skills, with the help of laparoscopic favorable vision exposure and magnification, it was not only safe but also preferential to carry out LPD combined with vascular resection and reconstruction, in addition, venous vascular invasion is not an absolute contraindication to LPD.

In 2016, Khatkov et al. (24) came to a study in which they launched eight cases of LPD combined with SMV/PV resection and reconstruction with four cases of wedge resection, one case of patch reconstruction, one case of end-to-end anastomosis, and two cases of artificial vascular replacement. They noted that LPD in combination with major venous resection is safe and feasible even in the case of longitudinal vein invasion. In 2018, Cai et al. (25) reported 18 cases of LPD combined with vascular resection and reconstruction with preferential approach of superior mesenteric artery, including eight cases of wedge resection, six cases of end-to-end anastomosis after surgery, and four cases of artificial vascular transplantation reconstruction. In their study, one case was transferred to OPD due to uncontrolled splenic venous hemorrhage. The average surgical time was 448 (420–570) min; the mean blocking time was 32 min, resection for 17 min, end-to-end anastomosis for 28 min, and artificial graft reconstruction for 48 min. Three patients developed biochemical fistula, one case had intraperitoneal hemorrhage after subcutaneous injection of LMW heparin and was stopped by conservative treatment. Overall, LPD combined with major venous resection and reconstruction is safe and feasible, and the anterior approach superior mesenteric artery priority approach can shorten both the operation time and occlusion time. Moreover, Cai et al. (9) reported one case of LPD combined with major venous resection accomplished by reconstruction through round ligament, and the postoperative recovery was good. Dokmak et al. (26) published one case of LPD with peritoneal parietal transplantation with PV reconstruction. Parietal peritoneal grafts are easier to obtain than artificial grafts, are not limited in size, or require long-term anticoagulation. Nevertheless, we did not take venous resection and reconstruction by virtue of hepatic round ligament reconstruction or peritoneal parietal transplantation reconstruction due to empirical limitations.

Because of the extremely high requirement of LPD combined with vascular resection, we believe that LPD combined with venous resection and reconstruction should be carried out on the basis of rich experience both in OPD combined with vascular resection and LPD. Our center has carried out OPD combined with vascular resection for more than 10 years. After completing more than 200 standard LPD cases (13), we selectively carried out LPD combined with venous resection. It has been reported that arterial resection and reconstruction combined with tumor resection may increase the incidence of postoperative complications and mortality and fail to improve patients’ survival (27). However, in our study, there was a patient who had tumor resection combined with right hepatic artery resection, the postoperative recovery was good, no complications occurred, and the tumor did not progress during the 9-month follow-up after the operation.

Ramacciato et al. (28) proposed that the first dissection of SMA during OPD allowed early detection of arterial invasion, avoided palliative resection, and then improved the R0 resection rate. In later literature reports, a variety of arterial priority approaches have been proposed, such as anterior, superior, uncinate, superior mesenteric approaches, etc. In clinical practice, it is reported that SMA preferential approach can not only detect early arterial invasion and avoid palliative tumor resection but also facilitates retroperitoneal lymph node dissection and reduces intraoperative blood loss. If the tumor invades the superior mesenteric vein or portal vein, combined phlebectomy increases the safety of surgery. In our report, SMA right approach was used in all cases, because the right approach can clarify the relationship between the tumor and SMA at an early stage, avoiding the occurrence of unresectable tumors after the pancreas neck is severed and early detection of variant arteries in the process of free arteries to avoid vascular damage. In addition, the right-side approach can dissect 16 groups of lymph nodes early, which is very important for the evaluation and impact of the patient’s prognosis. Moreover, the right approach preserves the celiac nerve plexus on the left side of the SMA, which can reduce the possibility of postoperative diarrhea. In the cases we reported, one patient had biochemical fistula, one patient with artificial venous transplantation and reconstruction had gastrointestinal hemorrhage after using anticoagulant drugs, and recovered after conservative treatment. There were no 30 days of death or re-admissions.

If SMV is involved in the confluence of the splenic vein (SV) and portal vein, complete resection of the tumor should be accompanied by the confluence vascular resection to facilitate the end-to-end anastomosis after phlebectomy, but whether the reconstruction of SV and SMV is controversial. At present, reconstruction is mostly advocated to avoid left portal hypertension and possible gastrointestinal bleeding caused by the obstruction of SV reflux after surgery. However, the reconstruction of SV often shifts the anastomosis to the left due to tension problems, which affects the smoothness of SMV and PV, as the gain outweighs the loss, it is not appropriate to force SV reconstruction. Whether the left portal vein hypertension and gastrointestinal hemorrhage occur after surgery still depends on the compensation of the collateral circulation, which is uncertain. In addition, after segmental resection of SMV or PV, if there is tension in vascular reconstruction, the ascending colon and small intestine retroperitoneum can be loosened, and the colon and small intestine can be rotated counterclockwise along the mesangial root to reduce the tension of the anastomosis. Dissection of the gastric coronary vein can also relieve the anastomotic tension, but if the severed SV is not reconstructed, the gastric coronary vein can be used as a compensatory vein to relieve the left portal hypertension after operation, so it should be retained as far as possible. In the cases of SV transection reported by Cai et al., no complications such as portal hypertension, gastrointestinal hemorrhage, or hypersplenism occurred, which were still observed in long-term follow-up. In this study, no cases of tumor invading confluence were involved.

The right-side entry of SMA shortens the specimen resection time, and when handling the tumor invasion of SMV-PV, it can be more convenient and safe to perform veins resection and anastomosis, which improves the R0 resection rate. Specific implementation steps are as follows: first, clear 8 and 12 groups of lymph nodes, disconnect the gastroduodenal artery and common bile duct, open the Kocher incision to the Treitz ligament and push the entire specimen to the left. The separation process reveals the inferior vena cava, left renal vein, and abdominal aorta. Then SMA roots were found between the left renal vein and the inferior vena vein, and remove the lymph nodes of both group 16 and SMA roots. From the SMA roots, free the right mesopancreatic pancreas along the arterial vascular direction. Then pull the head of the pancreas to the right, free the lower edge of the pancreas, expose the SMV, transcribe the pancreatic neck, and then reserve a blocking band at the SMV and PV, and the venous blood flow is also fully controlled, then it can be safely and calmly to free and remove and anastomosis or replace SMV or PV, which can significantly reduce complications and facilitate the recovery of patients.

At present, we have accumulated some experience of OPD with artificial vascular replacement, most patients have recovered well after the operation, and the PFS has improved without increasing the incidence of postoperative complications. It is worth noting that two cases of LPD combined with artificial venous replacement have been collected in this study, both of them had good short-term prognosis. Therefore, it is necessary to strengthen the collection of such cases for in-depth study in the future.

## Conclusion

LPD combined with major venous resection and reconstruction with a preferential choice of SMA approach is safe and feasible, which can shorten surgical time, improve R0 resection rate, and benefit patients’ short-term survival. However, this study only collected a few cases and just performed a 2-year follow-up, further accumulation of cases and long-term survival follow-up outputs is required in the later stage.

## Data Availability

The original contributions presented in the study are included in the article/Supplementary Material, further inquiries can be directed to the corresponding author/s.
